# Responding to COVID-19: New Trends in Social Workers’ Use of Information and Communication Technology

**DOI:** 10.1007/s10615-020-00780-x

**Published:** 2020-11-24

**Authors:** Faye Mishna, Elizabeth Milne, Marion Bogo, Luana F. Pereira

**Affiliations:** Factor-Inwentash Faculty of Social Work, University of Toronto, 246 Bloor St W, Toronto, ON M5S 1V4 Canada

**Keywords:** Information and communication technology, COVID-19, Paradigm shift, Impact, Clinical practice, Ethical boundaries, Client accessibility

## Abstract

COVID-19 changed the context for Information and Communication Technology (ICT) use globally. With face-to-face practice restricted, almost all communication with clients shifted to ICTs. Starting in April 2019, we conducted semi-structured interviews with social workers from four agencies serving diverse populations in a large urban centre, with the aim of exploring social workers’ informal ICT use with clients. Approximately 6 weeks after the cessation of face-to-face practice in March 2020 due to COVID-19 measures, we re-interviewed social workers (n = 11) who had participated in our study. Second interviews were based on a newly developed interview guide that explored social workers’ use of ICTs with clients in the context of COVID-19. Analysis of transcribed interviews revealed that the context of COVID-19 had generated two main themes. One, *a paradigm shift* for social workers was characterized by (a) diverse ICT options, (b) client-driven approach, and (c) necessary creativity. The second theme entails the *impact* of this transition which involved (a) greater awareness of clients’ degree of access, (b) confidentiality and privacy, and (c) professional boundaries. We discuss these themes and sub-themes and present implications for practice and research in a Post-COVID-19 world.

## Introduction

Information and Communication Technologies (ICTs) permeated direct social work practice long before COVID-19. ICTs include mobile devices (e.g., smartphones, tablets), computer hardware/software and other communication media (e.g., social media, text messaging). In addition to the adoption of ICTs to deliver formal service (e.g., e-counseling, tele-psychology) (Boydell et al. 2014), social workers were increasingly using ICTs informally with clients to communicate between sessions, as an adjunct to face-to-face practice (Mishna et al. 2012, 2014, 2015, 2019). In our recent survey, the majority of social workers in Canada (78.1% [n = 2034]), the U.S. (79.6% [n = 975]), Israel (74% [n = 285]), and the U.K. (86.9% [n = 106]) used ICTs informally with clients (Mishna et al. 2019). COVID-19, however, changed the context for social workers’ ICT use around the world. With the suspension of all non-essential face-to-face social work (OCSWSSW 2020) social workers are, in effect, now relying on ICTs for all work and communication with clients. The purpose of the current study, consequently, was to explore the use of ICTs by social workers with clients in the context of COVID-19.

## ICT Use in Clinical Practice

Prior to COVID-19, ICTs impacted clinical practice in three distinct ways: formal, blended, and informal ICTs (Mishna et al. 2017). Formal Online ICTs were depicted as standalone ICT programs/interventions, such as e-counseling and telepsychology (Boydell et al. 2014; Chan and Holosko 2016). In this model, online communication is the single mode of service provision, substituting for face-to-face practice. Using clear protocols, communication is conducted through designated software with security protections (Baker et al. 2014) and/or mobile phone applications and messaging services (Lee and Walsh 2015). In the second type of ICT use, referred to as formal blended ICTs, ICTs are integrated through a blending of online elements with face-to-face practice (van de Wal et al. 2015). Online exercises, such as homework assignments and psycho-educational activities are implemented to replace some face-to-face sessions (van der Vaart et al. 2014). Both online and face-to-face components are structured and monitored by a service provider (van de Wal et al. 2015). ICTs entered direct practice through informal (sometimes unpredictable or unsanctioned) use by social work practitioners as an adjunct to traditional face-to-face service. The primary (and formal) modality is face-to-face (Mishna et al. 2012, 2014). Informal ICT use occurs in conjunction with face-to-face practice through email, texting, or social media/social networking, and can be asynchronous or synchronous. Not meant to substitute for face-to-face practice, rather, informal ICT use typically serves as an unplanned added component. Reasons for informal ICT use range from practical purposes (e.g., scheduling) to complex discussions that are central to treatment goals (e.g., reporting intense distress).

## COVID-19: ICT Use in Clinical Practice

Without preparation, the COVID-19 pandemic and associated risk of contamination brought the need to practice physical distancing around the globe, which required significant changes in the way most services operate (Galea et al. 2020). On March 24, 2020, Ontario Canada ordered the mandatory closure of all non-essential workplaces to fight the spread of COVID-19 (Office of the Premier 2020). In response to the restrictions to face-to-face practice, governments, regulatory bodies and licensing boards in the United States and Canada temporarily relaxed restrictions surrounding ICT use and even encouraged mental health professionals to use new technologies with their clients (Wallace et al. 2020). For example, the Ontario College of Social Workers and Social Service Workers (OCSWSSW 2020) recommended that members consider providing services through any electronic device (e.g., a computer, tablet, smartphone, landline) or format (e.g., Internet, email, social media, chat, text, video).

Due to the rapid onset of COVID-19 restrictions, the shift to electronic service provision happened very quickly (Doorn et al. 2020; Olwill et al. 2020; Razai et al. 2020; Walter-McCabe 2020). With virtually no notice, social workers were required to transition to using ICTs to replace face-to-face services and all communication with clients. Without proper training or support on providing this treatment (Doorn et al. 2020), social service providers are left with many questions about how they can appropriately use technology to bridge the gap caused by COVID-19 (Wright and Caudill 2020). Nevertheless, social service providers have demonstrated a great deal of creativity in their use of technology to deliver services and maintain relationships with clients, and to provide continuity of care, necessary emotional support and communication (Boahen 2020; Farkas and Romaniuk 2020; Galea et al. 2020).

With this new context of COVID-19, the previous distinctions between informal and formal ICT communication have come into question. The traditional conceptualization of informal ICT use, as an adjunct to face-to-face service, no longer exists due to the cessation of face-to-face practice. Moreover, while governments, agencies, regulatory bodies, and licensing boards have asked social workers to provide their formal services remotely, this ICT use does not directly fit within the traditional definition of “formal ICTs”. The new ICT use encompasses a significant range of security protection and a lack of clear protocols.

## The Current Study

The current study is an amendment of a qualitative study initiated prior to the COVID-19 pandemic. The original study is the second of two sequential phases in a mixed-methods study. The first phase entailed an online survey administered to social workers in Canada, the United States, Israel, and the United Kingdom, comprising questions related to the frequency, nature and scope of informal use of ICTs in their traditional face-to-face practice. Beginning in April 2019, the second phase consisted of semi-structured interviews with social workers and clients. The phase one survey data informed the interview guide. The original guiding research questions examined the ways social workers used informal ICTs and the impact of this use on face-to-face practice. The onset of COVID-19 and the cessation of face-to-face practice, however, dramatically changed the context of social workers’ ICT use. With social workers forced to quickly transition to using ICTs for all work with clients, we recognized the need to explore social workers’ ICT use with clients in the context of COVID-19. Accordingly, we developed an additional interview guide to be utilized in the current study. The amended research questions investigate the ways social workers used ICTs during this global pandemic and the impact of this use on practice.

## Methods

The original qualitative study was amended and received approval on April 21, 2020 from the University of Toronto Health Sciences Research Ethics Board, to investigate social workers’ ICT use with clients in the context of COVID-19. Approximately 6 weeks after the cessation of face-to-face practice, we re-interviewed social workers (n = 11) who had participated in the study before their agencies implemented remote work. The second interviews were semi-structured phone interviews approximately 30 to 45 min in length.

### Recruitment and Sample

Recruitment for phase two of the study began in April 2019. Social work practitioners were recruited from four agencies across the Greater Toronto Area. These sites were selected to maximize variability in the sample as the participating agencies serve diverse populations of youth and adults with a range of individual, family and group-level services. To be eligible, service providers were required to: (1) be registered with the Ontario College of Social Workers and Social Service Workers or have a BSW or MSW; and (2) be involved in direct face-to-face practice.

For initial participant recruitment, agency administrators sent email invitations and flyers to introduce the study to individuals and provide the Research Coordinator’s contact information. In addition, research assistants set up recruitment tables in agency foyers and attended staff meetings to publicize the study. Twelve social work practitioners agreed to participate. Informed consent was obtained prior to either their in person or phone interviews. Participants were compensated with a gift card. Recruitment for the additional interviews during COVID-19 entailed the Research Coordinator sending email invitations and reminders to the social workers who had participated in the original interviews (n = 12). Participants did not receive additional compensation.

The data *corpus,* i.e., *all* data collected for this study, includes a total of 52 interviews with staff (n = 31) and clients (n = 21) (Braun and Clarke 2006). As data collection is ongoing, these numbers are expected to grow. The data *set,* i.e., data from the corpus being used for this analysis, comprises 11 social workers (9 females, 1 male, 1 Non-Binary/Gender Independent/Two-Spirit/Gender/Queer/GenderFluid/Agender) who agreed to participate in a phone interview during COVID-19. This data set was identified with a particular analytic interest: social workers affected by COVID-19 measures. The participants were consequently conducting most of their work remotely.

Participants were between 27 and 49 years, with an average of 31 years. Most participants had been in practice for 1–5 years (n = 5) or 6–10 years (n = 4), with one participant in practice for under 1 year, and one for over 20 years. Participants worked in diverse practice areas including mental health, developmental services, child welfare, violence against women, seniors, children, youth and families, case management, and 2SLGBTQ + therapy.

### Data Collection and Analysis

Individual interviews were conducted following the new semi-structured interview guides. Interview guides for the additional interviews explored the changes due to COVID-19 that had taken place with regards to social workers’ use of ICTs with clients, as well as opportunities and challenges introduced by these changes. Trained research assistants conducted the approximately 30–45-min telephone interviews. With participant consent, the interviews were audio-recorded using ID numbers, and transcribed verbatim by a transcription service to allow for thematic analysis of content (Braun and Clarke 2006). A list of matching ID numbers and names was compiled to ensure anonymity in the audio recording and transcripts. NVivo qualitative software was used to organize the data.

Research team members interpreted the data using Braun and Clarke’s recommended procedure for thematic analysis (2006). To provide an overall description of social workers’ use of ICTs during COVID-19, we decided to provide a rich thematic description of the entire data set, meaning that the themes identified, coded, and analysed would reflect the content of the entire data set. Furthermore, we chose to conduct inductive thematic analysis, by identifying themes that were most strongly linked to the data themselves. The themes were chosen based on the frequency with which they appeared across the data set, rather than the relevance to the researchers’ theoretical interests. Finally, we chose to frame the thematic analysis using a realist framework by deriving meaning in a straightforward way, whereby language was assumed to reflect and enable one to articulate motivations, experience and meaning. Thematic analysis, thus, used a semantic approach, in which themes were identified within the explicit meanings of the data, i.e., what a participant said (Braun and Clarke 2006).

Researchers, then, followed Braun and Clarke’s (2006) six step approach to engaging with text-based data, and began the coding process by reading and re-reading the interview transcripts to become familiar with the data. Next, using NVivo, researchers coded interesting features of the data in a systematic fashion across the data set, and collated codes into potential themes. During a series of meetings with the Principal Investigator, the research team reviewed the themes and began defining and naming the themes. An initial thematic map was created, which was further refined and reworked until the candidate themes were considered to adequately capture the contours of the coded data. Following the semantic approach, the initial description of the patterns in the semantic content of the data was followed by an interpretation of the broader meanings and implications (Braun and Clarke 2006).

### Findings

Analysis revealed two main themes, with three sub-themes each. The first theme, *a paradigm shift in ICT use,* encompassed three sub-themes, (a) diverse ICT options, (b) client-driven approach, and (c) necessary creativity. The second theme, *impact*, encompassed three interconnected sub-themes, (a) greater awareness of clients’ degree of access (b) confidentiality and privacy, and (c) professional boundaries.

### Theme One: A Paradigm Shift in ICT Use

All the participants’ descriptions of their changes to service delivery due to COVID-19 physical distancing measures indicated a paradigm shift in social workers’ ICT use. With the absence of face-to-face treatment, social workers and agencies introduced diverse ICT options, and adapted their ICT use to fit the needs of clients and creatively maintain the therapeutic relationship and process.

#### Sub-theme A: Diverse ICT Options

The social workers reported that their agencies’ responses to COVID-19 involved a substantial expansion of ICT options which social workers could use with clients. The participants all discussed their agencies’ new authorization to use either their work or personal cellphones as a method of providing phone counselling, in addition to several video-conferencing platforms through which to communicate with clients. Most agencies supported Zoom, FaceTime and/or WhatsApp video calling as platforms to provide formal sessions. Participants from only one agency reported being restricted to a single video-conferencing platform, OnCall, which is both HIPAA (Health Insurance Portability and Accountability Act) and PHIPA (Personal Health Information Protection Act) compliant. Most participants further reported their agencies’ new authorization of SMS, text messaging and/or WhatsApp messaging with clients, using either their work or personal cellphones. Participants from one agency reported their agency’s authorization of new uses of their existing email to communicate client care plans and safety plans, and as on participant explained, “whereas one might consider email an [informal] ICT, … we have found a way to include email as part of the therapeutic communication … We formalized it.”

In addition to the new dependence on ICTs for communication with clients during COVID-19 and in response to their agencies’ new authorization of ICT use, all participants reported adopting new ICT practices in their work with clients. Despite approval to use video-conferencing platforms, many reported introducing phone calls for many of their formal sessions with clients. Several participants, however, recounted using video-conferencing platforms, specifically Zoom or OnCall, as their main platform, whereas others used email and text messaging as their formal treatment. According to one participant, “if [text-messaging or email] are the only form of communication we can do in that moment, then I definitely provide that emotion coaching piece. So, validation, emotional support, practical support, as well as if they need behavioral coaching, I would provide that over email.”

Most participants relayed that they introduced new uses of ICTs with clients. A few social workers reported now using WhatsApp and text messaging to fulfill practical needs, which they previously had limited to face-to-face interactions, such as receiving documents from clients and sending resources to clients. One participant clarified, “before [COVID-19], they had to come to take the letter, come all the way to my office and let me see it. With text messages, they can just take me a picture and then I can do the translation for them.” Another participant stated, “I think a lot of the time what I originally used to arrange was an in-person session and then a follow-up phone call. Now that I’m doing a phone call session, I’m often doing a follow-up text, just to give that kind of space to let clients process and check in as they need.” In this instance, the participant’s original use of the phone call as an informal ICT communication method became their formal method of treatment during COVID-19, and text messaging between phone call sessions became the new informal ICT. These examples are illustrative of a paradigm shift in ICT use, as they demonstrate social workers’ introduction of new ICT uses, and devices, that go beyond traditional definitions of “formal” vs. “informal” ICTs.

#### Sub-theme B: Client-Driven Approach

A key component of the paradigm shift in ICT Use during COVID-19 consistently mentioned by social work participants was centering their practice based on the needs and preferences of clients. Many social workers described their agencies’ newfound flexibility towards staff’s selection of ICTs. For example, one participant explained, “they’ve just said, you know, as long as the person’s comfortable and you’re comfortable with them seeing where you live or whatever background you have then go ahead.” With respect to their agency’s preference for a particular type of ICT, another participant similarly observed, “they’ve sort of left it to our discretion. They’ve opened the conversation saying that no matter how we want to approach it they’re open to talking about it.” This flexibility was combined with encouragement of staff to choose ICTs based on the clients’ preferences. As one participant claimed, “as long as the client initiates and they give the consent of using that platform, well, [the social workers] still have to do it.” Another participant relayed, “we try to be client-centred, so whatever makes sense for the client in that moment. We are providing that option for each family. Comfort levels might be different for each family, so we have questions we ask families before starting the videoconferencing.”

Participants reflected this flexibility and client-driven approach. The majority described choosing ICTs based on the client preferences. For example, according to one participant, “I just do whatever works for the foster parents, I use WhatsApp, Zoom, FaceTime. Then with my teens, they don’t often want a video chat, so I talk to them on the phone or I text with them.” Another participant observed, “some families, some weeks are not really liking the whole phone, video piece, so it allows them to have a different form of therapy or guidance or support in times of need. Having those platforms as options has really allowed me to meet the families where they’re at and what they’re needing at that moment.” Participants demonstrated an awareness and flexibility based on the unique circumstances and needs of each client. As one social worker reflected, “I’m choosing to use email because it gives them more flexibility on when they respond. My families are home now with their kids which makes it harder for them to spend like 20 min talking to me on the phone because their kids are needing them. So, text and email are sometimes better.” Another participant similarly stated: “some parents have expressed that, because they have younger children with high needs, being able to set up a tablet or a laptop and sit in front of it for an hour might be challenging. Having the phone, being able to talk to me and move around to do things to keep their kids’ emotions managed and keep them busy has been helpful. Yeah, we’ve been really leaving it up to the client.”

#### Sub-theme C: Necessary Creativity

The third sub-theme within the theme of a paradigm shift in ICT use was the need for social workers to use creative strategies to remotely maintain their therapeutic relationships with clients. Many of the participating social workers reported finding innovative ways to sustain their connection with clients during these uncertain times. For example, one social worker observed, “at the beginning, I can see my contact drop, just very brief, much shorter interviews.” They explained that they then found new ways to connect with clients by texting or emailing them various activities or websites. This social worker further specified, “there was one website, they are doing a 13-day challenge using arts, and then I can do it together with the client, and we don’t need to talk that much, but somehow, we can stay connected in that way.”

Several participants described scenarios in which they exhibited creativity to maintain the therapeutic process despite the challenges of remote work. for example, a social worker depicted having trouble connecting with a client who was struggling to get out of bed due to depression. Remembering that this client was talented and enjoyed playing piano, the social worker suggested that the client play a song on the piano for the social worker over FaceTime. The social worker proposed to the client: “don’t talk and just play a song for me, not every day, but every other day, and then just three minutes, we can do it on Facetime, and after the three minutes we’re gone, we’re done, don’t say anything, just connect.” The client agreed and according to the social worker, told them that their regular musical FaceTime sessions gave the client “a reason to get out of bed, brush their teeth and wash their hair.”

A second participant who identified as an 2SLGBT + therapist observed that a few of their clients who were trans turned their camera off during some video sessions out of a desire not to be seen and not to see their own self-view on the screen. The social worker supported these clients in turning their cameras off during sessions. To ensure that nothing was lost in the session, the social worker explained that they were “by default, slowing down the session, checking in more often, using … not necessarily different language, but being very intentional about what language [they] use to check in.” The social worker further added that in response to no longer being able to read visual cues, such as their client’s body language or facial expressions, the social worker relied on their somatic training to check in with the client’s sensations, behaviors, imagery and meaning-making.

A third instance entailed a social worker who discussed the ways in which using ICTs as their formal treatment necessitated that they consider new ways of applying their clinical skills. They explained that in video-conferencing sessions, there is “a different type of intimacy” because of their ability to see each other’s homes. They specified the considerations they accordingly made: “someone might be more willing to share things over the computer that they wouldn’t necessarily be willing to share in-person.” The participants observed that while in a face-to-face session, they would be more confident about following the client’s lead, with web-based technology, they were mindful of the possible disinhibition effect of the internet (Suler 2004). Another participant said, “I realize I have to slow down and make sure to check in with them as to whether that is something that they, in fact, want to explore at this point.”

### Theme Two: Impact

In addition to describing the paradigm shift in the use of ICT during the COVID-19 context, social workers relayed the impact of this transition.

#### Sub-theme A: Greater Awareness of Clients’ Degree of Access

Most participants described the ways in which the transition to remote work during COVID-19 created a shift in client access to services. While some clients experienced enhanced access to services, others had less. This is illustrated by the following quote: “if you only ever offer services in one way, it would be inaccessible to someone. While switching to web-based is inconvenient for some, it’s incredibly convenient for others. We’re reaching slightly different client populations.” Several participants mentioned that clients who experienced anxiety were more able to access services during COVID-19. According to one participant, “some clients who previously had anxiety about going out are finding this actually, right now, is at least helping their anxiety to be able to stay at home and talk about what’s going on.” Participants noted greater access for clients in remote locations, as well as for clients who experience barriers to service, such as transportation issues. A social worker in an Indigenous agency explained that before COVID-19 their cultural programming was not accessible to their youth in foster care because they lived outside of the city. “When it became virtual,” however, they “saw how many of our kids in care were attending over Zoom,” and consequently “always made a separate one for our kids in care.” Finally, participants described the ways in which the transition to online services increased access for clients, particularly youth, who rely on others, such as their parents or guardians, to attend treatment. As one participant stated, “One client who I was having trouble scheduling with because their parents didn’t want to bring them, or they had to navigate their parents’ schedule, well, now, all of a sudden, it’s easier because they can just open their iPad and then, click, there I am.”

Most of the social workers recounted gaining awareness of the many ways in which this new reliance on ICTs to communicate with clients underscored some clients’ lack of access. The majority discussed difficulties contacting client based on the inequity of client resources, due to lack of access to Internet or smartphones. For instance, one participant observed, “the ones who don’t have Internet are definitely feeling a lot more isolated. They’re not able to participate in a lot of the online hangouts happening.” According to another participant, “I think one piece that can be challenging is being able to send resources, or anything to follow-up after sessions. Some clients don’t necessarily have internet at home. I have a few clients who only have it on their phone, so to be able to read or access a resource that way isn’t as useful as at the end of a session when you’re handing them a resource.” Of note, participants from two agencies reported that their agencies delivered cellphones to clients who did not have access to phones, each explaining that the agency was temporarily paying for the text and call capabilities.

Many participants commented on encountering challenges connecting with clients equally due to clients’ lack of computer literacy skills or comfort level with technology. One participant mentioned, “the families who are more computer literate definitely have an advantage in terms of being able to access resources and things like that because some things are really hard to explain over the phone and can’t be explained over text.” Another participant stated, “I work with seniors and not to say that seniors are not tech savvy, but the ones I’m connected with are for the most part choosing to just use phone or aren’t very comfortable with the Zoom technology.”

Many participants identified the difficulty contacting clients due to a client’s lack of privacy, especially those living with perpetrators of abuse or with family members who do not support their therapy. One participant stated, “some clients, for sure, there’s an inability to find a private space, as well, at home because of being at home with family. We deal with some cases of elder abuse, in which case clients live with potentially the abuser. In those cases, it can be really challenging to support clients in the same way as we would have if they were able to come into the office to talk about what’s actually going on.” Another participant explained that a client whose family did not support their therapy, faced additional scheduling difficulties as they shared a computer with siblings.

#### Sub-theme B: Confidentiality and Privacy

Participants stressed the impact of the shift to online treatment and increased ICT use, on their ability to protect clients’ confidentiality and privacy. Most participants maintained such concerns despite reporting that their agencies had put in place new guidelines to uphold clients’ privacy. For example, one participant stated, “even though I have conversations with families prior to using email and text messages around the confidentiality, I think at times of crises, of panic or high emotions, those guidelines are dismissed.” Another participant noted, “some clients, I find, are maybe sharing more over email than they normally would because they don’t always have the time or space to phone, so that can be challenging to navigate. We do tell people that our email isn’t necessarily confidential, but people don’t have the space at home to be able to pick up the phone and call because of work situations, people at home with them.” One participant found it challenging to protect client privacy due to their own lack of private space: “Your roommate maybe hears when you’re talking to your client, so that might be my concern. But our practice is we try to minimise … we share the common area to talk, I will go back to my room and talk. But still there is the chance other people can hear the conversation. The privacy is not like before COVID-19 because of the setting. Everybody has to stay home, and everybody’s home settings are different.”

#### Sub-theme C: Professional Boundaries

Most participants described encountering more difficulties maintaining professional boundaries with clients, which they attributed to COVID-19 and their agencies’ new flexibility towards ICT use. One participant explained, “I am more prone to checking my emails outside of work hours now that I’m working from home and even respond to emails outside of work hours, whereas before I would generally not do that.” Another participant remarked that their clients, “know I’m working from home, so they don’t understand why I can’t just write them back all the time or answer my phone all the time.” According to another social worker, “It was a lot clearer for people to understand, oh, she’s in the office 9 to 5, Monday to Friday. I think that’s been made a bit more blurry. And it’s probably harder for people to keep track of the days of the week.” With respect to clients contacting them outside of work hours, one participant relayed, “yeah, because before, we were never receiving any text messages, but here and there, every now and then, I will receive a text message maybe on a weekend or at night.” Some participants associated their issues in maintaining boundaries with having provided their cellphone numbers to clients. One participant compared giving clients their cellphone number prior to and during COVID-19: “Some of them did, some didn’t. Yeah, that’s interesting, like, I would just, kind of, choose who I gave it to. But now I have given it to everyone.” Despite having anticipated potential boundary issues, other participants observed that providing their cellphone number did not cause such issues.

## Discussion

Due to the urgent and drastic restrictions to deter the COVID-19 virus, agencies and practitioners had no choice but to rapidly alter service delivery from in-person to online (Doorn et al. 2020; Olwill et al. 2020; Razai et al. 2020; Walter-McCabe 2020). The current study is unique in its exploration of social workers’ transition from face-to-face treatment to remote practice during COVID-19. The findings revealed two major themes: a paradigm shift in ICT use; and the impact of COVID-19 on practice.

### Paradigm Shift in ICT Use

Most participants reported that, to ensure continuity of services for clients, their agencies had rapidly introduced new ICT options. Such guidance on ICT use during COVID-19 echoes the literature. Recognizing the need to accommodate the exceptionally rare circumstances of the COVID-19 pandemic, agencies, governments and regulatory bodies temporarily suspended strict requirements related to the use of online platforms (Barsky 2020; Walter-McCabe 2020; Wright and Caudill 2020), and encouraged social workers to switch to virtual methods of treatment to serve those in need (Farkas and Romaniuk 2020; NLASW 2020; OCSWSSW 2020). As a result, some technologies that are more easily accessible to clients such as FaceTime, Google Hangouts, and Facebook video chat could now be used if necessary (Barsky 2020; Wright and Caudill 2020). Furthermore, in a recent UK study (Cook and Zschomler 2020), social workers reported replacing their face-to-face sessions with virtual sessions through FaceTime, WhatsApp, Skype, Google Hangouts, Microsoft Teams and Zoom following the COVID-19 lockdown. As many clients have limited access to technology with more secure communications, this temporary introduction of easily accessible ICT options allowed social workers to avoid “abandoning clients in need” (Barsky 2020, p. 2).

In addition to introducing diverse ICT options, agencies and practitioners showed impressive commitment to core social work values. CASW Guidelines for Ethical Practice (2005) require that “social workers maintain the best interests of clients as a priority” (p. 3), and the NASW’s value of service can be interpreted as making “the needs and wellbeing of the client paramount” (Farkas and Romaniuk 2020, p. 70). As a result, agencies and social workers upheld the value of client-centered care in their ICT use, by prioritizing client preferences and needs.

Social workers showed creativity in their use of diverse ICTs to maintain the therapeutic process. As an example, by encouraging a depressed client to regularly play piano for a few minutes on FaceTime, one participant provided mirroring of the client’s intense depression and difficulty functioning and of the client’s enjoyment and talent (Kohut 1984; Melano Flanagan 2016). Similar demonstrations of social workers’ creativity during the pandemic can be found in the literature (Cook and Zschomler 2020; Farkas and Romaniuk 2020). For example, in Cooke and Zschomler’s (2020) study, a social worker described choosing a ‘Minecraft’ backdrop for their video call as an innovative tool to capture the interest of their younger clients. Social workers’ creativity and innovation is considered essential to overcoming the challenges associated with this global pandemic (Farkas and Romaniuk 2020; Galea et al. 2020), and responding to complex problems more generally (Eadie and Lymbery 2007).

Through such innovative and flexible use of ICTs, social workers were able to maintain their therapeutic relationship with clients. A foundational tenet of social work practice is the centrality of the relationship with the client (i.e., individual, family or group) (Bogo 2018), and the importance of the relationship on therapeutic outcomes (Horvath 2001; Wampold 2015). Our findings contradict concerns that digital counseling options could dilute the meaning of the therapeutic relationship and alliance (Reamer 2015). One social worker described the ways in which the ability to observe one another in their own homes through videoconferencing enhanced the intimacy of the therapeutic relationship. This was similarly found in Mitchell’s (2020) study, whereby clients explained that sharing their home environments can create a shift in power balance and thus a more equal relationship. Another social worker discussed how the option to turn the camera off during video sessions allowed social workers to comply with the wish of clients who were trans to not be seen. Likewise, Cook and Zschomler (2020) found that during COVID-19 social workers reported that text and instant messaging enhanced client openness to talk about issues that they did not feel comfortable discussing in a face-to-face setting. This is consistent with findings of Mitchell’s (2020) study that, to fit the attachment needs of the client, therapists and clients would collaboratively select the “online arrangement of what is seen and not seen” (p. 127). While ICTs pose some challenges to the therapeutic process, such as technological interruptions or intrusion of privacy (Cipolletta et al. 2017), it is important to acknowledge the positive opportunities that ICTs can create for the therapeutic alliance (Mitchell 2020).

### Impact

Analysis of the interviews revealed significant impacts of the COVID-19 context on social work practice. First, the participants reported becoming increasingly aware of the inequity in clients’ ability to access services, both pre- and post COVID-19. Clients who had previously experienced barriers to participating in face-to-face service due to factors such as experiencing extreme anxiety, living in remote locations, or relying on others for transportation, were more able to access services with the transition to remote sessions. In contrast, clients with fewer digital resources and digital literacy skills experienced barriers to accessing services, although they may not have found it challenging to access face-to-face treatment. Cook and Zschomler (2020) similarly found that digital exclusion, in the form of limited Internet and inability to pay data costs, was an obstacle to children and families accessing services during COVID-19. As the context of COVID-19 is expected to increase problems related to anxiety, depression, loneliness, fear, substance use, and domestic violence and abuse (Galea et al. 2020; Razai et al. 2020; Zhou et al. 2020), the finding that the social workers are increasingly cognizant of clients’ inequitable access to treatment is critical. This finding is consistent with other studies in which therapists have expressed concerns regarding dealing with clients’ insufficient Internet literacy, in addition to clients’ existing vulnerabilities related to poverty, rural settings, older age, and health inequities (Doorn et al. 2020; Razai et al. 2020; Walter-McCabe 2020). As the pandemic has “sharpened this digital divide” (Farkas and Romaniuk 2020, p. 1), and exposed existing inequities, there is greater need for advocacy (Farkas and Romaniuk 2020).

A further impact of the paradigm shift on social work participants was due to their own and the agencies’ flexibility towards ICT use during COVID-19. The overall flexibility towards the type and structure of ICT communication created ethical dilemmas. First, while providing diverse ICT options enhanced some clients’ access to services, participants stressed the impact of this shift on their ability to protect clients’ confidentiality and privacy. Specifically, many of the platforms approved in response to COVID-19 are not HIPAA or PHIPA compliant (Barsky 2020; HIPAA Journal 2017; Farkas and Romaniuk 2020; Miliard 2020). Although several agencies encouraged social workers to explain the possible risks to client confidentiality and privacy prior to using a platform, some participants noted that when clients are in distress, they may ignore these risks. Such circumstances, therefore, create a dilemma for social workers as they are required to both prioritize the preferences of their clients, and protect their clients’ right to privacy and confidentiality. These concerns are mirrored in the literature’s analysis of agency responses to COVID-19 (Farkas and Romaniuk 2020), and generally correspond with a discussion in which agencies and professionals have been engaging regarding ethical issues related to confidentiality and the use of ICTs as a means of communication with clients (Walter-McCabe 2020).

A second dilemma stems from the new flexibility towards the structure of ICT communication. When reflecting this client-centered approach, many participants described frequently changing the method and timing of their ICT communication based on the needs of each client. Combined with new agency policies surrounding ICT use, such as the requirement for social workers to provide their work cellphone numbers to clients, this flexibility created difficulties for participants in their navigation of professional boundaries with clients. Some participants, for example, observed that clients began to text and phone social workers outside of work hours including weekends. This linking of additional boundary challenges by the participants is supported in the literature, which discusses the greater likelihood of boundary issues associated with providing services after typical office hours, in the evenings and on weekends, due to remote work during the pandemic (Barsky 2020; Martin et al. 2020). Reamer (2015) discusses the ways that the transition from a very structured, controlled environment of face-to-face service to working from home can create ambiguities for clients and thus confuse practitioner-client boundaries.

## Limitations

This study contains several limitations. The small sample size suggests the need to be cautious in generalizing to social workers, although all but one of the initial participants agreed to be re-interviewed. Nevertheless, social workers with different experiences may offer other insights and views. Another limitation is the absence of client participants, which is required to fully grasp the impact of COVID-19 on the use of ICTs. Despite these limitations, the current study provides a unique opportunity to examine social workers’ ICT use with clients in real time during the COVID-19 pandemic. Moreover, the results are supported by the existing literature.

## Implications for Practice

In a remarkably short time, social workers responded to COVID-19 by fundamentally changing their communication with clients. With the absence of face-to-face practice, previously the most common formal or primary mode, a paradigm shift in ICT use rapidly took place. In the immediate short-term, governments, agencies, regulatory and licensing boards, and social workers responded with both flexibility and suggestions for caution. While social workers are encouraged to respond with a client-centered approach and creativity to ensure client wellbeing and maintain connections with clients, this new integration of ICTs in practitioners’ practice poses additional challenges for social workers (Barsky 2020; OCSWSSW 2020).

Two key challenges that have emerged during COVID-19 are paradoxical. On the one hand is the need to explore integrating HIPAA or PHIPA-compliant apps (e.g., doxy.me) in formal service to protect clients’ privacy/confidentiality. On the other hand, there is the need to promote clients’ equal access to services beyond the context of COVID-19, which may be hindered by the integration of HIPAA or PHIPA-compliant ICTs in all formal service (Barsky 2020; Farkas and Romaniuk 2020; Walter-McCabe 2020). While evidence emerged of agencies responding to client accessibility needs during the pandemic, such as delivering cellphones to clients, the question of whether and how advocacy and support can be sustained in a post-COVID-19 world must be considered. As the increased integration of ICTs in social work practice will likely continue once face-to-face practice resumes, agencies and social workers will be faced with the challenge of reducing the barriers to clients’ ability to access ICTs in the long-term. It is thus suggested that agencies and social workers advocate for clients’ equal access to online services, beyond the context of the COVID pandemic (Walter-McCabe 2020). A challenge is for social workers to address these competing demands.

A major implication of the switch to increased ICT use consists of participants’ greater difficulties navigating professional boundaries with clients. In both the short and long-term, it is necessary to manage these challenges. Social workers are advised to use risk reduction strategies to prioritize client safety, and agree on the boundaries of communication prior to beginning service (e.g., expectations regarding response time, social media use) (Barsky 2020; Martin et al. 2020). Social workers are further directed to engage in self-care, such as turning off their phone when not on-call (Hansel 2020). Importantly, Cook and Zschomler (2020) found that social workers’ wellbeing during COVID-19 was positively affected by informal supports provided by their colleagues, such as virtual check ins and text-based communication. There is a need to consider and evaluate additional strategies to help social workers navigate these challenges to maintaining their boundaries with clients.

## Implications for Research

Prior to COVID-19, there was a lack of research on informal ICT use by social workers with clients (Mishna et al. 2014, 2019). Our findings indicate that due to the pandemic there has been a paradigm shift in the ways social workers use ICT, with diverse ICT platforms having entered social work treatment. Research is needed on this markedly changed use of ICTs in the COVID-19 context, particularly as there is currently no or little primary or “formal” face-to-face treatment. Research should examine how ICT use will evolve over time and whether the previous distinctions (i.e., formal, informal ICT use) remain relevant. It will be critical to determine the processes through which practitioners select ICT platforms (e.g., PHIPA compliance, client preferences and needs) and the implications for clients (e.g., degree of access, privacy). On an ongoing basis, there is a need for researchers to make sense of this changing social work digital landscape, and to evaluate the implications of these changes for clients as well as for social workers.

As the world grapples with a ‘new normal’ in moving towards post COVID-19, it is incumbent on social work to consider the paradigm shift in practice and the impact on social work.
